# Biological variation in serum thyroid, iron metabolism, and plasma bone metabolism biomarkers in patients with type 2 diabetes mellitus

**DOI:** 10.3389/fendo.2025.1506664

**Published:** 2025-05-20

**Authors:** Xia Wang, Mei Zhang, Zhi Liu, Chuan Li, Yujue Li, Hengjian Huang, He He, Xijie Yu

**Affiliations:** ^1^ Department of Laboratory Medicine, West China Hospital, Sichuan University, Chengdu, China; ^2^ Department of Thoracic Surgery, West China Hospital, Sichuan University, Chengdu, China; ^3^ Department of Nuclear Medicine, Sichuan Provincial People’s Hospital, School of Medicine, University of Electronic Science and Technology of China, Chengdu, China; ^4^ Department of Endocrinology and Metabolism, Laboratory of Endocrinology and Metabolism, Rare Disease Center, West China Hospital, Sichuan University, Chengdu, China

**Keywords:** biological variation, type 2 diabetes mellitus, thyroid, iron, biomarker

## Abstract

**Objectives:**

Variability in biomarkers is crucial for clinical decision-making in individuals with type 2 diabetes mellitus (T2DM). The biological variation (BV) of biomarkers associated with thyroid function, iron metabolism, and bone metabolism may show population-specific differences. This study aims to evaluate the biological variation of sixteen biomarkers in T2DM patients and compare these with variations observed in a healthy population.

**Methods:**

Twenty-four T2DM patients, aged 43 to 67 and in stable condition, were enrolled. Blood samples were collected biweekly for three months. Analysis of variance models were used to assess the BV, including within-subject BV (CV_I_), between-subject BV (CV_G_), analytical variation, reference change value (RCV), index of individuality (II), the number of samples required for steady-state set points (NHSP), and analytical performance specifications for all biomarkers.

**Results:**

Females exhibited lower CV_I_ estimates for thyroid-stimulating hormone, parathyroid hormone, and phosphate compared to males. No significant differences in CV_I_ estimates were observed between T2DM patients and healthy individuals across the study. However, the CV_G_ estimates for cortisol and iron were significantly lower in T2DM patients compared to the healthy individuals.

**Conclusions:**

BV data is critical for the precise interpretation of serial biomarker level changes in T2DM patients. It is deemed reasonable to use RCVs for four bone metabolism markers and five thyroid biomarkers, derived from a healthy population, as a reference for monitoring T2DM patients.

## Introduction

Type 2 Diabetes mellitus (T2DM) is a prevalent health issue that has significantly grown over the past few decades, becoming a significant challenge to public health worldwide (1). There are strong associations between diabetes mellitus and numerous concurrent health issues, such as osteoporosis, thyroid dysfunction, and abnormalities in iron metabolism (2–4). Biomarkers can be used for diagnosis, risk stratification, and management of complications in patients with T2DM. Therefore, it is important to accurately interpret variations in these biomarker results, which are influenced by biological variation (BV) (5, 6).

Biomarker variation refers to the fluctuation of an analyte around a homeostatic set point (HSP) and encompasses both individual and analytical variations (7). While improvements in analytical techniques and testing processes can reduce analytical variation, individual variability is likely influenced by specific populations and may differ across various epidemiological studies (8). Most studies on BV focus on healthy populations, with few examining individuals with T2DM. Previous studies have explored BV in conditions such as kidney transplantation, chronic liver disease, and heart failure (6, 9, 10), revealing that BV data from healthy individuals often differs from those in unhealthy populations. Consequently, our study aims to investigate the BV of an expanded set of biomarkers in patients with T2DM.

Biomarkers for thyroid function are essential in diagnosing and managing thyroid-related disorders. T2DM can lead to a decrease in thyroid-stimulating hormone (TSH) levels and impair the transformation of thyroxine (T4) to triiodothyronine (T3) in the peripheral tissues (11). There are individual variations in thyroid hormones related to factors such as age, circadian rhythms, and hypothyroidism (12–14), but there are no data on BV in patients with T2DM. Similarly, patients with T2DM frequently experience disturbances in bone and mineral metabolism (15). Common measurands of bone metabolism include calcium (Ca), phosphate (PHOS) and parathyroid hormone (PTH), and 25-hydroxyvitamin D [25(OH)D]. Moreover, high iron is a risk factor for T2DM (16), and biomarkers are used to assess iron homeostasis. We included four common metrics, serum iron, transferrin saturation (TSAT), unsaturated iron-binding capacity (UIBC), and total iron-binding capacity (TIBC), and the variability of these metrics in patients with T2DM was not known previously.

We analysed BV data for sixteen serum/plasma biomarkers, including four related to bone metabolism, four to iron metabolism, five thyroid biomarkers, and three additional hormones in patients with T2DM. These data were used to determine the reference change value (RCV), the number of samples needed for steady-state set point (NHSP), and the analytical performance specifications (APS). Ultimately, we compared these results with previously published data from healthy populations.

## Materials and methods

### Participants and samples

Patients with T2DM were enrolled in the study following an eligibility assessment based on specific inclusion and exclusion criteria. The inclusion criteria were as follows: First, male and female participants aged 18 to 70 were eligible; secondly, participants diagnosed with T2DM without complications for at least three months, according to the American Diabetes Association guidelines published in 2015; thirdly, participants needed to have been on a stable diabetes medication regimen for at least 3 months prior to enrolment; fourthly, participants had to be capable of understanding the study requirements and providing written informed consent. The exclusion criteria included: firstly, individuals treated with insulin, vitamin D supplements, or medications that affect thyroid function; secondly, participants with severe, uncontrolled comorbid conditions within the last three months; thirdly, participants with severe psychological disorders that may interfere with their ability to comply with study procedures; and fourthly, women who were pregnant, breastfeeding, or planning to become pregnant during the study period. This study received approval from the Institutional Ethical Review Board of the West China Hospital of Sichuan University (No. 20201079). Each participant voluntarily signed an informed consent form after being informed about the content and purpose of the study.

Fasting venous blood samples were collected (BD Vacutainer^®^, New Jersey, USA) biweekly between 8:30 a.m. and 9:30 a.m., a total of six times. Plasma tubes were centrifuged within 45 min at 3000 g for 10 min at 4°C, while serum tubes were centrifuged at 22°C. Serum and plasma were then stored at −80°C until analysis.

### Analytical methods

Quantitative determination of serum TSH, T3, free triiodothyronine (FT3), T4, free thyroxine (FT4), cortisol (CORT), insulin (INS), C-peptide (C-P), plasma intact PTH, and 25(OH)D was performed using the Roche Cobas e601 (Roche, Basle, Switzerland) with immunoassay electrochemiluminescence reagents and calibrators. The assays for TSH, T3, T4, and CORT were conducted utilising first-generation reagents, whereas FT3 and FT4 assays used third-generation reagents. INS, C-P, and intact PTH tests employed second-generation reagents. Serum PHOS, iron, Ca, and UIBC were analysed using Roche Cobas 8000 (Roche, Basle, Switzerland). Total iron-binding capacity (TIBC) is calculated as the sum of Serum Iron and UIBC, and TSAT is determined by the ratio of serum iron to TIBC. All samples were measured in duplicate in a single run.

### Data analysis

To obtain analytical variation (CV_A_) and within-subject biological variation (CV_I_) estimates, data were analysed using standard ANOVA or CV-ANOVA. The CV-ANOVA method is based on the CV transformation, which normalises the data for each individual by dividing by the mean value of each individual (17). The CV_G_ estimates were calculated by a standard nested ANOVA after identifying outliers between subjects with Reed’s criterion and the Dixon-q test2 (18). Outliers from replicates and within-subject were excluded using the Bartlett test and the Cochran test. The Shapiro-Wilk test was used to analyse data normality, and log-transformation was applied to non-normally distributed data. The steady state of subjects was assessed by linear regression of six pooled mean group sample concentrations for each biomarker (19). Subjects were considered to be in a steady state when the 95% confidence interval (CI) of the regression line’s slope included zero. Mean values and BV estimates were calculated for the entire study population and separately for women and men. The 95% CI for BV estimates was calculated using Miller’s formula (20). Differences in mean values and BV estimates between subgroups were considered significant if the 95% CI did not overlap. The CV_I_ and CV_G_ values for the entire study population were applied to APS using the criteria: CV = 0.5CV_I_; B = 0.25 (CV_I_^2^ + CV_G_^2^)^0.5^; total allowable error (TE) = 1.65CV + B. The RCV, index of individuality (II), and the NHSP were calculated for each measurand according to the following formula:


$$\text{RCV\%}=100\text{\% }\times \left(\text{exp}\left(\pm \sqrt{2}\times \text{Z}\times \sqrt{{\text{CV}}_{\text{I},\text{ln}}2^{\;}+{\text{CV}}_{\text{A},\text{ ln}}2\;}\right)-1\right)$$

$$\text{C}{\text{V}}_{\text{A},\text{ ln}}=\;{\left[\text{ln }\left(\text{C}{\text{V}}_{\text{A}}2^{\;}+1\right)\right]}^{0.5}$$

$$CV_{I,\;ln}=\;{\left[ln\;\left(CV_{I}2^{\;}+1\right)\right]}^{0.5}$$

$$\text{ II}=\sqrt{{\text{CV}}_{\text{I}}2^{\;}+{\text{CV}}_{A}2^{\;}}/$$

$$\text{ NHSP}={\left(\text{Z}\times \sqrt{{\text{CV}}_{\text{I}}2^{\;}+{\text{CV}}_{A}2^{\;}}/\text{D}\right)}^{2}$$

The Z factor was set at 1.96, indicating a two-sided change and a 95% probability. The D values represented deviations of 10%, 15%, and 20% from the true HSP (21).

For normally distributed data with equal variances, use mean ± SD and T-tests. Otherwise, use median and Kruskal-Wallis tests.

## Results

Twenty-four patients diagnosed with T2DM (11 men and 13 women), aged 43–69 years, were included in this study. We collected the medical and medication histories of each participant, including metformin, glimepiride, miglitol, gliclazide, and acarbose. All participants were non-smokers and non-alcohol drinkers. Of the 24 participants, 20 completed all six collections, and four completed four collections; the mean number of blood samples per participant was 5.7. Baseline characteristics and the concentrations of sixteen biomarkers for all participants, as well as for the men and women subgroups, are summarised in **Table 1**. All subjects showed no systematic changes in the concentrations of these biomarkers during the follow-up, as confirmed by linear regression (**Supplementary Table S1**). Reference intervals for each measurand are summarised in **Supplementary Table S1**, and all measurements fell within these defined ranges. Details about the outliers are provided in **Supplementary Table S2**. The median and 95% CI of eight hormones for each individual, grouped by sex, are shown in **Supplementary Figure S1**. The remaining eight measurements (including four for bone metabolism and four for iron metabolism biomarkers) are shown in **Supplementary Figure S2**.

**Table 1:** Baseline characteristics and concentrations of sixteen biomarkers for patients with T2DM, grouped by sex.

Sort	All participants	Males	Females	P value^a^
Number of participants	24	11	13	–
Total number of results	136	62	74	–
Total number of samples	272	124	148	
Age, year	55 (7)	54 (15)	56 (11)	0.64
BMI	55 (12.5)	54.5 (10)	56.0 (13.8)	0.64
FPG, mmol/l	6.52 (2.67)	4.98 (1.05)	5.11 (0.91)	0.39
HbA1C, %	6.5 (1.7)	6.85 (2.43)	6.3 (1.0)	0.09
TSH, mIU/L	2.18 (2.03)	1.94 (1.82)	2.5 (2.2)	0.32
FT3, pmol/l	4.41 ± 0.46	4.69 ± 0.49	4.18 ± 0.3	<0.001
FT4, pmol/l	15.9 ± 1.84	16.45 ± 1.65	15.45 ± 1.88	0.01
T3, nmol/l	1.54 ± 0.23	1.6 ± 0.27	1.5 ± 0.18	0.015
T4, nmol/l	93.93 (18.81)	96.03 (18.81)	90.8 (23.42)	0.005
CORT, nmol/l	287.88 ± 77.17	314.0 ± 67.73	264.49 ± 77.82	<0.001
INS, uU/ml	6.11 (3.6)	6.57 (3.73)	6.33 ± 2.7	0.02
C-P, nmol/l	0.63 ± 0.15 (0.15)	0.65 ± 0.13 (0.19)	0.62 ± 0.17	0.31
PTH, pmol/l	4.92 (1.02)	4.92 (2.03)	5.31 ± 1.56	0.31
25 (OH)D, nmol/l	62.29 (26.97)	63.75 (29.19)	61.89 (16.26)	0.89
Ca, mmol/l	2.35 ± 0.07	2.34 ± 0.09	2.35 ± 0.07	0.45
PHOS, mmol/l	1.16 ± 0.13	1.12 ± 0.21	1.17 ± 0.1	<0.001
Iron, umol/l	16.6 (5.24)	17.1 (5.07)	16.48 (6.01)	0.07
UIBC, umol/l	37.07 ± 6.34	37.09 ± 7.83	35.64 ± 6.42	0.38
TSAT, %	31.92 (8.96)	32.3 (9.09)	30.78 (10.13)	0.32
TIBC, umol/l	53.56 ± 6.38	54.71 ± 7.48	52.52 ± 6.65	0.04

a The P value represents the comparison between males and females.

CI, confidence interval; FPG, Fasting Plasma Glucose; TSH, thyroid stimulating hormone; FT3, free triiodothyronine; FT4, free thyroxine; T3, triiodothyronine; T4, thyroxine; CORT, cortisol; INS, insulin; C-P, c-peptide; PTH, parathyroid hormone; 25 (OH)D, 25-hydroxyvitamin D; Ca, calcium; PHOS, phosphorus; UIBC, unsaturated iron-binding capacity; TSAT, transferrin saturation; TIBC, total iron-binding capacity.

No statistically significant differences were observed in the concentrations of TSH, C-P, PTH, 25(OH)D, Ca, iron, UIBC, and TSAT between genders. However, significant intersexual disparities were identified in the levels of FT3, FT4, T3, T4, CORT, INS, and TIBC, with males exhibiting markedly higher levels than those exhibited by females (*P* < 0.05). The only exception was the mean serum concentration of PHOS, which was significantly elevated in women compared to men (*P* < 0.05).

The results for CV_A_, CV_I,_ and CV_G_, along with their 95% CIs, for sixteen biomarkers are displayed in **Table 2**. The BV components of CV_I_ and CV_G_ based on healthy populations from the European Federation of Clinical Chemistry and Laboratory Medicine Biological Variation Database (EFLM BVD) are also presented (22). The reliability of the CV_I_ estimates was confirmed by SD_A_/SD_I_ ratios, according to the recommendations of Røraas et al. (23), with all biomarkers demonstrating ratios below the threshold of 1.0. According to the 95% CI, CV_I_ estimates for TSH, PTH, and PHOS calculated for females were lower than those derived for males. For the entire study population, CORT and iron CV_G_ estimates were significantly lower than those reported by the EFLM BVD, whereas the overall CV_I_ estimates were similar between patients with T2DM and healthy individuals.

**Table 2:** Biological variation estimates of CV_A_, CV_I_, and CV_G_, with 95% CIs, for sixteen biomarkers and compared against the EFLM BV database.

Biomarker	Sort	CV_A_, % (95% CI)	CV_I_, % (95% CI)	CV_G_, % (95% CI)	SD_A_/SD_I_	CV_I_, % (95% CI) EFLM BV database	CV_G_, % (95% CI) EFLM BV database
TSH, mIU/L	All articipants	1.6 (1.4-1.7)	18.2 (16.0-21.1)	42.9 (33.4-60.2)	0.1	17.9 (14.7-29.3)	36.1 (23.9-48.4)
	male		21.9 (18.2-27.4)	41.7 (29.2-73.2)			
	female		15.2 (12.9-18.6)	44.7 (32.0-73.7)			
FT3, pmol/l	All participants	1.2 (1.1-1.3)	4.5 (4.0-5.2)	9.5 (7.4-13.3)	0.3	5.1 (4.7-7.9)	8.1 (8.0-22.5)
	male		4.3 (3.6-5.3)	9.1 (6.3-15.9)			
	female		4.8 (4.1-5.8)	5.6 (4.0-9.3)			
FT4, pmol/l	All participants	2.6 (2.4-2.8)	5.3 (4.6-6.1)	10.4 (8.1-14.6)	0.5	4.8 (4.6-9.5)	8.0 (7.5-12.1)
	male		5.4 (4.5-6.7)	8.6 (6.0-15.1)			
	female		5.1 (4.4-6.3)	11.3 (8.1-18.7)			
T3, nmol/l	All participants	3.9 (3.6-4.3)	6.2 (5.4-7.1)	13.6 (10.6-19.1)	0.6	6.2 (5.1-10.4)	11.1 (4.4-20.4)
	male		6.2 (5.2-7.7)	16.3 (11.4-28.6)			
	female		6.2 (5.2-7.5)	10.2 (7.3-16.9)			
T4, nmol/l	All participants	1.7 (1.5-1.8)	5.8 (5.1-6.7)	15.1 (11.7-21.1)	0.3	6.4 (4.9-7.4)	11.8 (11.0-12.2)
	male		5.2 (4.4-6.6)	15.0 (10.5-26.4)			
	female		6.3 (5.3-7.6)	14.5 (10.4-24.0)			
CORT, nmol/l	All participants	2.4 (2.2-2.6)	19.4 (17.2-22.6)	18.8 (14.6-26.6)	0.1	16.1 (15.5-26.6)	33.6 (28.8-53.1)
	male		16.0 (13.5-20.2)	15.1 (10.5-26.4)			
	female		22.9 (19.3-28.1)	19.3 (13.7-32.7)			
INS, uU/ml	All participants	3.4 (3.1-3.7)	20.7 (18.3-23.9)	36.5 (28.3-51.1)	0.2	25.4 (21.1-37.1)	33.5 (31.5-81.8)
	male		19.1 (16.0-23.8)	36.1 (25.2-63.4)			
	female		22.5 (19.1-27.5)	37.5 (26.9-61.9)			
C-P, nmol/l	All participants	1.1 (1.0-1.2)	11.4 (10.1-13.3)	21.5 (16.6-30.4)	0.1	–	–
	male		12.0 (10.0-15.2)	16.7 (11.5-30.6)			
	female		10.9 (9.2-13.3)	25.2 (18.0-41.5)			
PTH, pmol/l	All participants	2.5 (2.3-2.8)	18.4 (16.3-21.2)	26.3 (20.5-36.9)	0.1	14.7 (11.3-25.9)	28.9 (21.8-43.3)
	male		21.9 (18.4-27.2)	27.4 (19.2-48.2)			
	female		15.2 (12.9-18.6)	26.1 (18.7-43.1)			
25 (OH)D,mol/l	All participants	2.7 (2.5-3.0)	6.9 (6.1-8.0)	23.7 (18.3-33.6)	0.4	6.8 (1.8-12.8)	30.1 (23.0-64.3)
	male		6.3 (5.2-7.8)	23.6 (16.5-41.5)			
	female		7.4 (6.2-9.0)	24.8 (17.6-42.1)			
Ca, mmol/l	All participants	0.8 (0.8-0.9)	2.1 (1.9-2.5)	1.6 (1.2-2.2)	0.4	1.8 (0.8-2.3)	2.7 (1.6-4.1)
	male		2.0 (1.7-2.5)	1.3 (0.9-2.2)			
	female		2.3 (1.9-2.7)	1.9 (1.4-3.1)			
PHOS, mmol/l	All participants	1.2 (1.2-1.4)	7.8 (6.9-9.0)	8.4 (6.5-11.8)	0.2	7.7 (5.7-8.3)	10.7 (7.9-17.4)
	male		10.1 (8.5-12.6)	8.8 (6.2-15.5)			
	female		5.6 (4.8-6.9)	6.4 (4.6-10.6)			
Iron, umol/l	All participants	2.2 (2.0-2.4)	19.1 (16.9-22.0)	9.7 (7.5-13.6)	0.1	27.6 (19.8-30.3)	26.7 (25.1-32.3)
	male		17.6 (14.8-21.9)	9.8 (6.9-17.2)			
	female		20.5 (17.4-24.9)	9.1 (6.5-15.0)			
UIBC, umol/l	All participants	1.3 (1.2-1.4)	10.1 (8.9-11.6)	14.7 (11.4-20.8)	0.1	–	–
	male		9.3 (7.8-11.7)	14.9 (10.2-27.2)			
	female		10.6 (9.1-13.1)	15.1 (10.8-24.9)			
TSAT, %	All participants	1.4 (1.3-1.5)	18.3 (16.2-21.2)	12.0 (9.3-17.0)	0.1	–	–
	male		17.1 (14.2-21.4)	13.0 (9.0-23.8)			
	female		18.8 (16.0-23.0)	11.2 (8.0-18.4)			
TIBC, umol/l	All participants	1.4 (1.3-1.5)	6.1 (5.4-7.1)	11.0 (8.5-15.6)	0.2	–	–
	male		4.6 (3.9-5.8)	9.9 (6.8-18.0)			
	female		6.8 (5.7-8.2)	11.1 (8.0-18.3)			

CV_A_, analytical variation; within-subject biological variation (CV_I_), between-subject biological variation (CV_G_); CI, confidence interval; SD_A_/SD_I_, ratio between analytical (SD_A_) and within-subject variance (SD_I_); TSH, thyroid stimulating hormone; FT3, free triiodothyronine; FT4, free thyroxine; T3, triiodothyronine; T4, thyroxine; CORT, cortisol; INS, insulin; C-P, c-peptide; PTH, parathyroid hormone; 25 (OH)D, 25-hydroxyvitamin D; Ca, calcium; PHOS, phosphorus; UIBC, unsaturated iron-binding capacity; TSAT, transferrin saturation; TIBC, total iron-binding capacity.

**Table 3** presents the RCV, II, and NHSP values for each biomarker. The APS were derived from the CV_I_ and CV_G_ of sixteen measurands for all participants. For INS, the NHSP could be estimated with 95% probability using eight samples if the D values were set at 15%. The II values for five thyroid hormones, INS, C-P, 25(OH)D, and TIBC, were <0.6, whereas the II estimates for iron, TSAT, and Ca were >1.4.

**Table 3:** Analytical performance specification and NHSP for sixteen biomarkers based on biological variation estimates.

Biomarker			NHSP			APS derived from present study		
	RCV, % (Decrease; Increase)	II, %	10%	15%	20%	Imprecision, %	B, %	TE, %
TSH, mIU/L	-39.7; 65.8	0.42	13	6	3	9.11	11.66	26.69
FT3, pmol/l	-12.1; 13.8	0.49	1	1	1	2.26	2.63	6.36
FT4, pmol/l	-14.9; 17.6	0.56	1	1	1	2.63	2.91	7.24
T3, nmol/l	-18.3; 22.3	0.53	2	1	1	3.08	3.74	8.81
T4, nmol/l	-15.3; 18.0	0.4	1	1	1	2.88	4.03	8.78
CORT, nmol/l	-41.8; 71.8	1.04	15	7	4	9.72	6.76	22.8
INS, uU/ml	-44.1; 78.74	0.58	17	8	4	10.37	10.49	27.6
C-P, nmol/l	-27.1; 37.3	0.53	5	2	1	5.71	6.08	15.49
PTH, pmol/l	-40.2; 67.1	0.71	13	6	3	9.21	8.03	23.22
25(OH)D, nmol/l	-18.4; 22.6	0.31	2	1	1	3.43	6.17	11.83
Ca, mmol/l	-6.1; 6.5	1.43	1	1	1	1.07	0.67	2.43
PHOS, mmol/l	-19.6; 24.5	0.94	2	1	1	3.92	2.87	9.34
Iron, umol/l	-41.2; 70.3	1.98	14	6	4	9.57	5.37	21.16
UIBC, umol/l	-24.5; 32.4	0.69	4	2	1	5.04	4.46	12.76
TSAT, %	-39.8; 66.2	1.53	13	6	3	9.17	5.48	20.61
TIBC, umol/l	-15.9; 18.9	0.57	2	1	1	3.06	3.15	8.2

RCV, reference change value; NHSP, number of samples required to homeostatic set point; APS: analytical performance specification; TSH, thyroid stimulating hormone; FT3, free triiodothyronine; FT4, free thyroxine; T3, triiodothyronine; T4, thyroxine; CORT, cortisol; INS, insulin; C-P, c-peptide; PTH, parathyroid hormone; 25(OH)D, 25-hydroxyvitamin D; Ca, calcium; PHOS, phosphorus; UIBC, unsaturated iron-binding capacity; TSAT, transferrin saturation; TIBC, total iron-binding capacity.

## Discussion

Currently, BV data from healthy individuals are widely used in clinical settings for many common measurands. To ensure the reliability of these data, the EFLM Biovariation Working Group published the BV Critical Appraisal Checklist (BIVAC) (24). According to the study criteria for BV data, this study provides the first-ever BV estimates and RCV for sixteen biomarkers in patients with T2DM, assessing whether differences between measurements and HSP are clinically relevant.

Thyroid hormones are crucial endocrine regulators influenced by multiple factors, including genetics, environment, disease status, and circadian rhythm (25). To date, BV studies on thyroid hormones have predominantly focused on healthy populations, with only a few examining patients with hypothyroidism or pregnant women (26, 27). Moreover, the sampling interval must be considered when studying BV in thyroid hormone (28). There are long-term BV studies of thyroid hormones and short-term BV studies that lasted one year and 24 h, respectively (14, 29). In this study, the CV_I_ and CV_G_ estimates for TSH, FT3, and FT4 in patients with T2DM are similar to those reported in the European Biological Variation Study, which is also a mid-term study (30). Meanwhile, patients with T2DM exhibited comparable CV_I_, CV_G_, and RCV estimates to those from another study focusing on the elderly population (31). In clinical practice, using the TSH RCV estimates (65.8%) from this study, an individual’s serum TSH concentration, initially measured at 2.0 mIU/L, could naturally rise to 3.3 mIU/L without any pathological cause, ascribed to the combined effects of biological and analytical variability. All five analytes used to assess thyroid function showed low II values (<0.6), indicating high individuality; similar findings were reported in a meta-analysis of BV in thyroid-related measures (32).

As another important endocrine hormone, CORT exhibits a more pronounced circadian rhythm than that of thyroid hormones and is affected by multiple factors, including season, disease, and sex (33). Compared to the EuBIVAS BV estimates for morning serum CORT in healthy populations, patients with T2DM showed higher CV_I_. However, similar CV_G_ values were observed in subgroups of men and women older than 50 years (34). Unlike other measurands, INS and C-P are integral to the pathology of T2DM patients, where insufficient INS secretion or INS resistance can lead to the development of the disease. Unfortunately, no BV meta-analysis results for C-peptide were available in the EFLM BVD. Dittadi R et al. (35) provided estimates of CV_G_ and CV_I_ for serum C-peptide in healthy individuals, which were higher than those found in this study; however, their data lacked corresponding confidence intervals for both CV_G_ and CV_I_. The EuBIVAS reported a higher CV_I_ estimate than what was observed in our study (36), a discrepancy that may be attributed to differences in health status among the study populations.

Parathyroid hormone and vitamin D are crucial regulators of Ca and PHOS and are widely used in diagnosing and treating bone metabolism disorders (37). This study analysed total 25(OH)D and intact PTH levels in plasma. Total 25(OH)D primarily comprises 25-hydroxyvitamin D_3_ [25(OH)D_3_], with its active form being 1,25-hydroxyvitamin D [1,25(OH)_2_D]. The plasma concentration of 25(OH)D varied up to 6–7 times among participants and was affected by factors such as diet, season, and genetics, and it does not remain constant over time (38). Consequently, Cavalier E et al. suggested that any APS derived from BV estimates may not be suitable for this parameter (39). However, in this study, the patients were in a stable state, and the CV_I_ and CV_G_ did not exhibit any significant differences compared to those in healthy individuals. The fluctuation in 25(OH)D concentration also affects PTH secretion, thereby influencing calcium and phosphorus homeostasis (38). Similar to 25(OH)D, PTH exists in multiple forms. Second-generation PTH assays measure not only the full-length, biologically active PTH 1–84 but also large C-terminal PTH fragments, which tend to accumulate in patients with chronic kidney disease. Corte Z and Venta R (40) assessed the BV estimates of PTH in haemodialysis patients and healthy individuals using the same analytical method employed in our study. Our CV_I_ estimates for PTH were higher than those reported for healthy subjects in their study, as indicated by the mostly non-overlapping 95% CI of CV_I_. However, BV estimates for PTH, Ca, and PHOS in this study were consistent with the meta-analysis results reported by EFLM BVD. A high II for Ca, exceeding 1.4, suggests a low degree of individual variation, implying that population-based reference intervals are expected to have good diagnostic sensitivity.

Here, we present the BV estimates for four markers used in the diagnosis of anaemia and iron metabolism. To date, the EFLM BV database includes fifteen studies on BV data for iron assays, with CV_I_ values ranging from 1.3 to 38.4%. This variation is largely attributed to differences in study duration. Iron overload or deficiency can lead to metabolic disorders (41), making it crucial to evaluate BV values for iron metabolic markers in such conditions. Unfortunately, only two studies have reported BV data for TSAT and TIBC. The CV_I_ estimates for TSAT were 25.9% and 38.2% (8, 42), which were higher than the 18.3% observed in patients with T2DM. This indicates a need for more studies to meet clinical requirements for interpreting iron metabolism biomarkers.

Overall, the BV data obtained from patients with T2DM in this study contribute to enriching the BV database, which is still under development. The BV data are essential for accurately interpreting changes in serially detected biomarker levels in patients with T2DM. The index of individuality (> 1.4) for Ca, iron, and TSAT indicates that the reference intervals are likely to exhibit good diagnostic sensitivity. The RCV, derived from BV data, is considered an optimal approach for monitoring patients with chronic conditions (43). Our findings show that BV data for these four bone metabolism analytes and five thyroid biomarkers in T2DM patients are similar to those in healthy individuals. This finding supports the rationale for applying RCVs developed using BV data from healthy individuals to patients with T2DM who are in a stable condition. It is worth noting that CV_A_ in this study was derived from repeated samples. Laboratories should estimate RCVs based on their specific conditions. Furthermore, a more precise analysis of BV is needed, as hormonal analytes are influenced by rhythms and seasons. This study mainly focused on patients over 50 years old, with only three patients between 40 and 50 years of age, highlighting a gap in BV assessment for younger patients. Future research should address these aspects.

## Data Availability

The original contributions presented in the study are included in the article/**Supplementary Material**. Further inquiries can be directed to the corresponding author/s.

## Supplementary material

Supplementary Figure 1 The median values and 95% CIs of thyroid biomarkers, CORT, INS, and C-P for each participant according to sex. CI, confidence interval; TSH, thyroid stimulating hormone; FT3, free triiodothyronine; FT4, free thyroxine; T3, triiodothyronine; T4, thyroxine; CORT, cortisol; INS, insulin; C-P, c-peptide.

Supplementary Figure 2 The median values and 95% CIs of bone metabolism and iron metabolism biomarkers for each participant according to sex. CI, confidence interval; PTH, parathyroid hormone; 25(OH)D, 25-hydroxyvitamin D; Ca, calcium; PHOS, phosphorus; UIBC, unsaturated iron-binding capacity; TSAT, transferrin saturation; TIBC, total iron-binding capacity.
