# Supplementary figures and images for: Biological variation in serum thyroid, iron metabolism, and plasma bone metabolism biomarkers in patients with type 2 diabetes mellitus
Source: Front Endocrinol (Lausanne). 2025 May 20;16:1506664. doi: 10.3389/fendo.2025.1506664 (PMC12129759; doi:10.3389/fendo.2025.1506664)

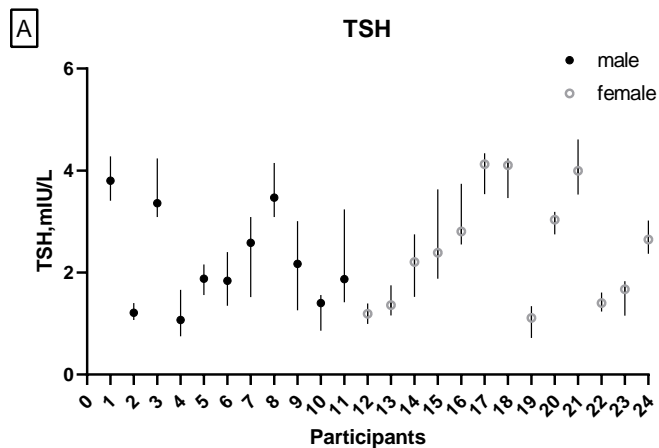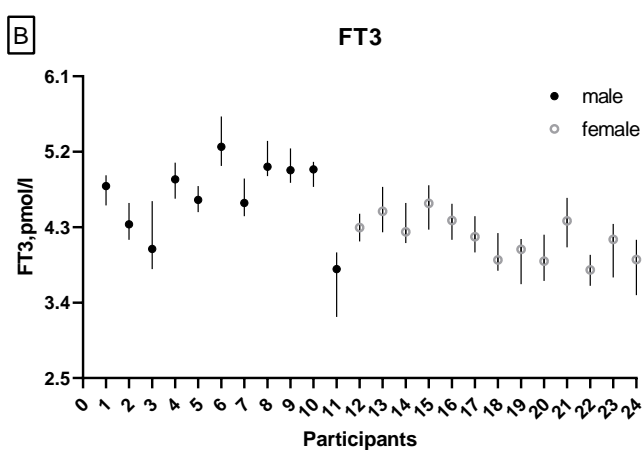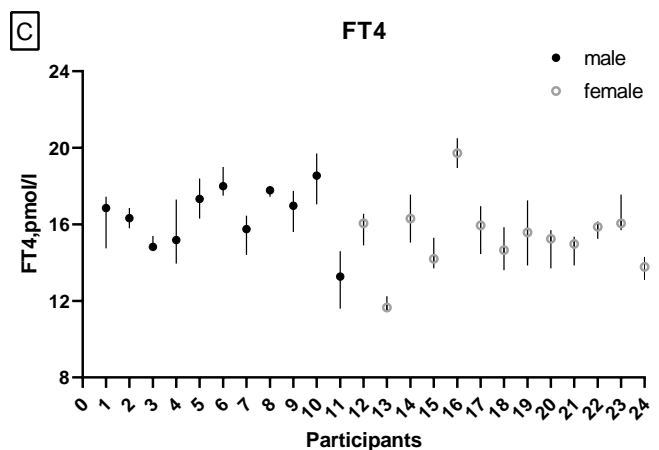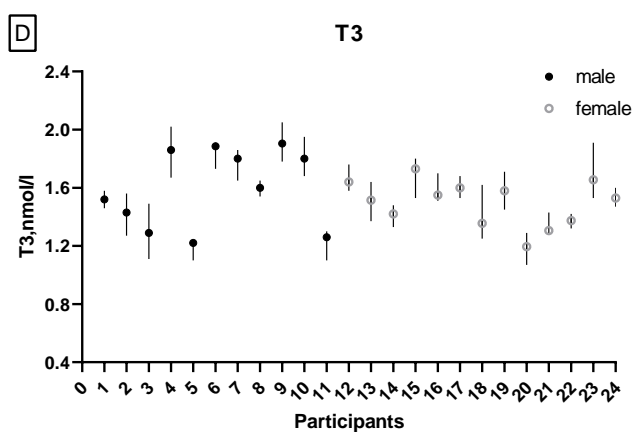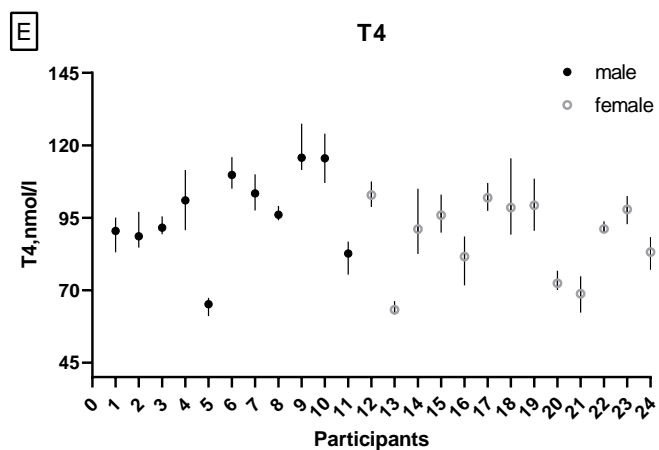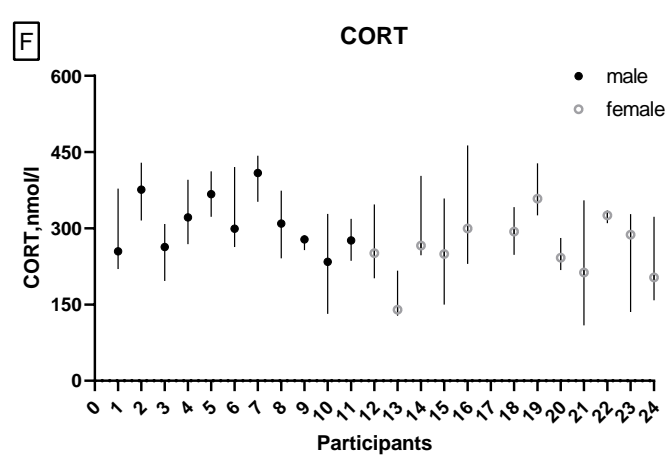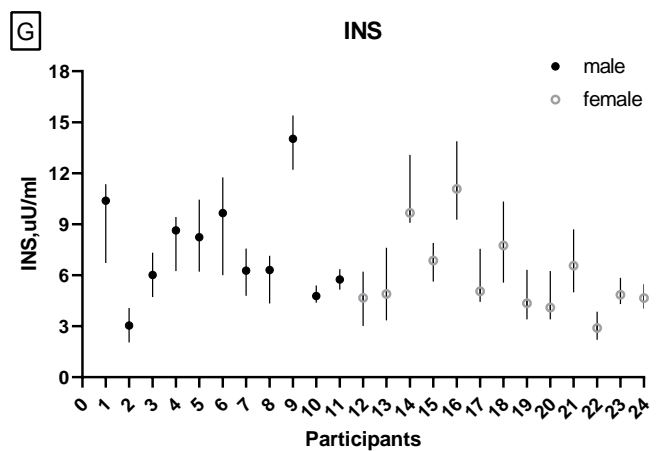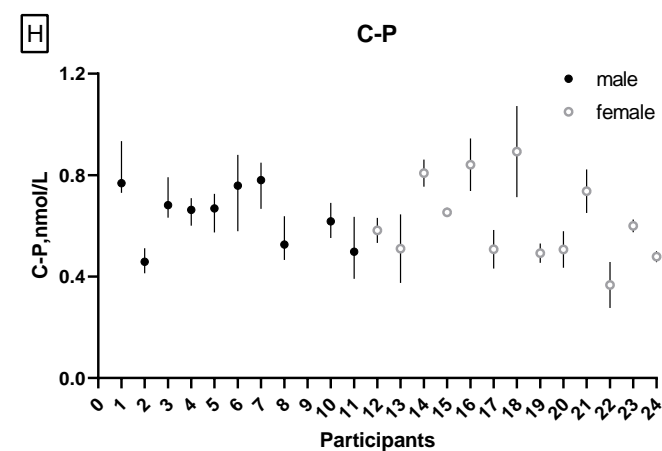

Supplement: Supplementary Figure 1 — The median values and 95% CIs of thyroid biomarkers, CORT, INS, and C-P for each participant according to sex. CI, confidence interval; TSH, thyroid stimulating hormone; FT3, free triiodothyronine; FT4, free thyroxine; T3, triiodothyronine; T4, thyroxine; CORT, cortisol; INS, insulin; C-P, c-peptide. [file DataSheet1.pdf]

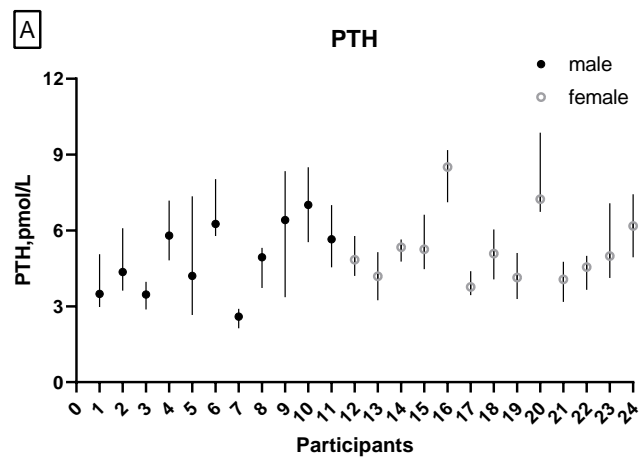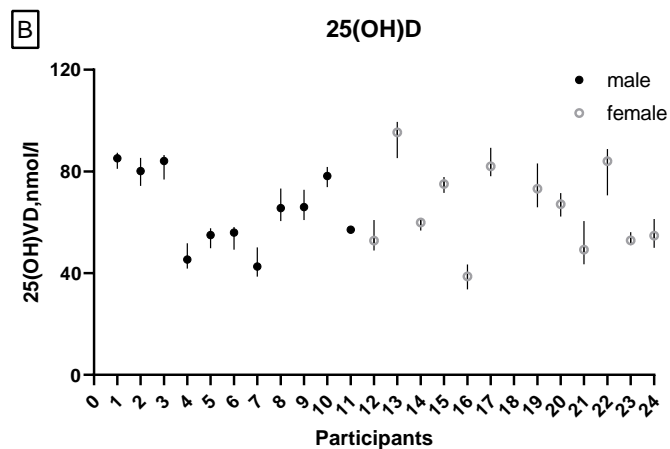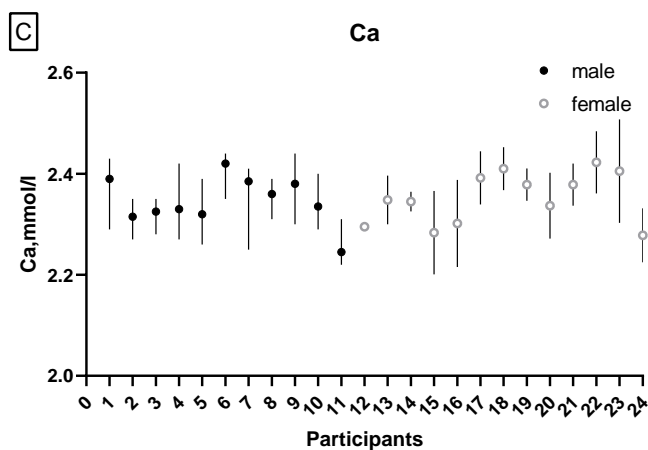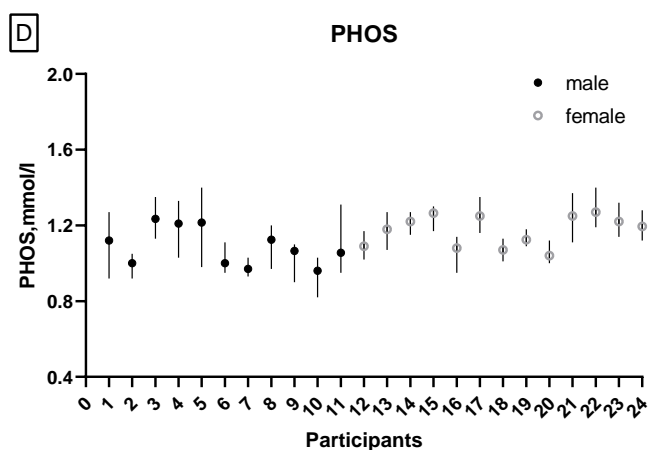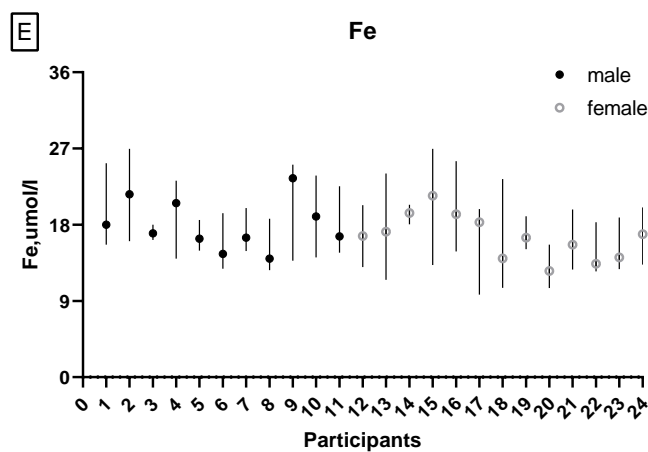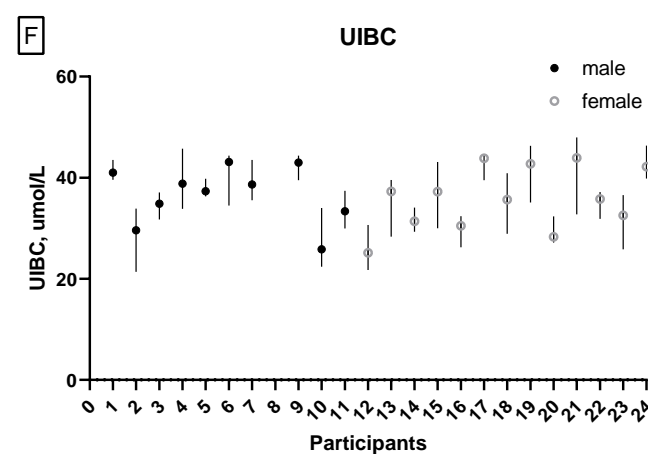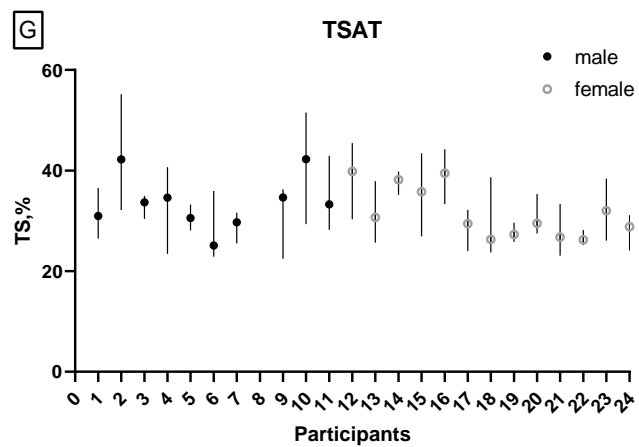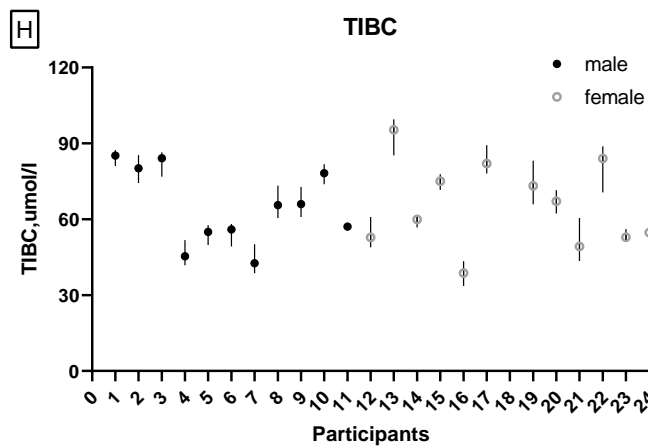

Supplement: Supplementary Figure 2 — The median values and 95% CIs of bone metabolism and iron metabolism biomarkers for each participant according to sex. CI, confidence interval; PTH, parathyroid hormone; 25(OH)D, 25-hydroxyvitamin D; Ca, calcium; PHOS, phosphorus; UIBC, unsaturated iron-binding capacity; TSAT, transferrin saturation; TIBC, total iron-binding capacity. [file DataSheet2.pdf]
