# Supplementary material for: Biological variation in serum thyroid, iron metabolism, and plasma bone metabolism biomarkers in patients with type 2 diabetes mellitus
Source: Front Endocrinol (Lausanne). 2025 May 20;16:1506664. doi: 10.3389/fendo.2025.1506664 (PMC12129759; doi:10.3389/fendo.2025.1506664)
Supplement: Supplementary file 3 [file Table1.docx]

Table S1: The 95% Confidence Intervals of the slopes of the linear regression on the six pooled mean group sample concentrations for each measurand and reference intervals for measurands.

| Biomarker | 95% Confidence Interval | Reference interval |
| --- | --- | --- |
| TSH | -0.13 ~ 0.41 | 0.27~ 4.20 mIU/L |
| FT3 | -0.26 ~ 0.99 | 3.6 ~7.5 pmol/l |
| FT4 | -0.17 ~ 0.15 | 12.0 ~ 22.0 pmol/l |
| T3 | 0.98 ~1.59 | 1.3 ~ 3.1nmol/l |
| T4 | -0.035 ~ 0.005 | 62 ~ 164 nmol/l |
| CORT | -0.005 ~ 0.002 | 133 ~ 537 nmol/L |
| INS | -0.1 ~ 0.1 | 1.5 ~ 15 uU/ml |
| C-P | -1.55 ~ 2.4 | 0.48 ~ 0.78 nmol/L |
| PTH | -0.17 ~ 0.19 | 1.6 ~ 6.9 pmol/L |
| 25(OH)D | -0.03 ~ 0.01 | 47.7 ~ 144 nmol/L |
| Ca | -3.8 ~ 5.8 | 2.11 ~ 2.52 mmol/L |
| PHOS | -1.5 ~ 3.1 | 0.85 ~ 1.51 mmol/L |
| Iron | -0.05 ~ 0.1 | Male：10.6 ~ 36.7 μmol/L  Female：7.8 ~ 32.2 μmol/L |
| UIBC | -0.05 ~ 0.05 | 19.7 ~ 66.2 umol/l |
| TSAT | -0.03 ~ 0.05 | 20 ~ 55 % |
| TIBC | -0.04 ~ 0.05 | 48.3 ~ 68.0 μmol/L |
| HbA1C | -0.3 ~ 0.04 | 4.0 ~ 6.0％ |
| FPG | -0.12 ~ 0.07 | 3.9 ~ 5.9 mmol/L |

Table S2: Number of outliers and test results used for ANOVA

| Biomarker | Number of outliers | | | Included results | | Number of outliers (%) |
| --- | --- | --- | --- | --- | --- | --- |
|  | Replicate  (Analytical) | Samples  (Within) | Subjects  (Between) | Subjects | Results |  |
| TSH | 2 | 7 | 0 | 24 | 256 | 5.8 |
| FT3 | 0 | 0 | 0 | 24 | 272 | 0 |
| FT4 | 0 | 3 | 0 | 24 | 266 | 2.2 |
| T3 | 0 | 4 | 0 | 24 | 264 | 2.9 |
| T4 | 2 | 3 | 0 | 24 | 264 | 2.9 |
| CORT | 2 | 8 | 1 | 23 | 254 | 6.6 |
| INS | 0 | 4 | 0 | 24 | 264 | 2.9 |
| C-P | 2 | 8 | 1 | 23 | 254 | 6.6 |
| PTH | 0 | 2 | 0 | 24 | 268 | 1.5 |
| 25(OH)D | 0 | 7 | 1 | 23 | 258 | 5.1 |
| Ca | 0 | 2 | 0 | 24 | 268 | 1.5 |
| PHOS | 2 | 2 | 0 | 24 | 266 | 2.2 |
| Iron | 0 | 0 | 0 | 24 | 272 | 0 |
| UIBC | 0 | 7 | 1 | 23 | 258 | 5.1 |
| TSAT | 0 | 10 | 1 | 23 | 252 | 7.4 |
| TIBC | 0 | 7 | 1 | 23 | 258 | 5.1 |
